# Wired for companionship: a meta-analysis on social robots filling the void of loneliness in later life

**DOI:** 10.1093/geront/gnaf219

**Published:** 2025-09-30

**Authors:** Fahimeh Mehrabi, Akram Ghezelbash

**Affiliations:** Department of Sociology, University of Calgary, Calgary, Alberta, Canada; Faculty of Economics and Business Administration, Ferdowsi University of Mashhad, Mashhad, Iran; Haskayne School of Business, University of Calgary, Calgary, Alberta, Canada

**Keywords:** Loneliness, Human-robot interaction, Artificial intelligence, Social support

## Abstract

**Background and objectives:**

Loneliness is a significant public health concern affecting over a quarter of older adults worldwide. Emerging research suggests that artificial intelligence (AI)-enabled social robots may offer a viable alternative for providing a new form of social support and reducing loneliness. This meta-analysis evaluates the effectiveness of AI-enabled social robots in reducing loneliness among older adults and examines the conditions under which these interventions are most effective.

**Research Design and Methods:**

A systematic search was conducted through October 2024. Effect sizes from 19 studies (*N* = 1,083) were synthesized using robust variance estimation (RVE) in meta-regression. Moderation analyses examined how social robots’ effectiveness differs by contextual factors such as participants’ backgrounds and studies’ characteristics.

**Results:**

Our findings indicated that social robots significantly reduced loneliness among older adults. However, studies with control groups indicate a higher effect size. Moreover, greater reductions in loneliness are observed among individuals in institutional settings compared to those living independently. In addition, stronger intervention effects reported in Japan and Turkey than in the United States. However, age, cognitive status, robot type, duration of intervention, and year of publication did not significantly influence intervention effectiveness.

**Discussion and Implications:**

Findings underscore the potential of social robots as an effective and scalable approach for addressing loneliness among older adults, particularly within institutional care environments. Policymakers, gerontologists, and care providers should consider integrating AI-enabled social robots into existing care frameworks, emphasizing culturally sensitive and inclusive implementation strategies.

Loneliness, also referred to as the “geriatric giant,” affects over a quarter of older adults worldwide, with its prevalence increasing as individuals age (Duffner et al., 2024; Susanty et al., 2025). Substantial evidence links feelings of loneliness in later-life to heightened risks of chronic illnesses, premature mortality and severe mental health challenges such as suicidal ideation (Cené et al., 2022; Wong et al., 2024). As global populations age and traditional social support systems face mounting strain, the need for innovative, scalable strategies to support older adults’ psychosocial well-being has become urgent (Seckman, 2023). In recent years, artificial intelligence (AI)-enabled social robots—autonomous or semi-autonomous technologies designed to engage users in interactive social behaviors—have gained increasing attention as potential interventions to mitigate loneliness (Henschel et al., 2021; Pu et al., 2019). Through programmed behaviors, verbal communication, and nonverbal cues that simulate human or pet-like presence, social robots are designed to function as a form of social support (Thalmann, 2022). However, despite increasing interest, questions remain regarding the overall effectiveness of social robots and the specific contexts and populations in which they are most beneficial.

This meta-analysis addresses these gaps by systematically evaluating the effectiveness of social robot interventions in reducing loneliness among older adults. Specifically, this study aims to quantify the overall impact of social robots on loneliness and examine how their effectiveness varies according to participant characteristics (e.g., living arrangement, cognitive status, etc.) and intervention characteristics (e.g., robot type, intervention duration, etc.). Understanding these dynamics is critical not only for advancing theoretical knowledge about human-robot relationships but also for informing the design and implementation of interventions aimed at promoting well-being in aging populations. By synthesizing and analyzing the existing body of quantitative research, this study offers crucial insights into when, where, and for whom social robots may serve as effective tools to combat the “geriatric giant.”

## Background

As older adults undergo key life transitions, such as retirement and declining health, their social networks often contract, leaving them increasingly vulnerable to loneliness (Mümken et al., 2024). Although interventions like group activities, cognitive-behavioral therapies, and animal-assisted programs aim to reduce loneliness, they frequently fall short for individuals facing mobility challenges, cognitive impairments, or limited access to community resources (Gardiner et al., 2018; Mushtaq & Khan, 2024). In response, some emerging evidence suggests that AI-enabled social robots can foster emotional connections with older adults and reduce feelings of loneliness by providing a consistent source of interaction that mimics companionship (Chen et al., 2020; Laban et al., 2024). These robots vary widely in their design and functionality, ranging from physically embodied robots equipped with sensors, cameras, and actuators that enable them to respond to user interactions (e.g., robotic cats) to voice-based agents (e.g., Amazon Alexa) that use speech recognition and natural language processing to engage in conversation (Bansal et al., 2024). Regardless of their form, social robots share a fundamental aim: to provide older adults with dependable, socially engaging interactions in contexts where traditional forms of support may be unavailable.

The potential of social robots to reduce loneliness is best understood through well-established psychosocial frameworks. Drawing on general theories of loneliness, social support is most valuable when it provides emotional reassurance, consistent interaction, and a sense of belonging (Drageset, 2021). While human relationships traditionally fulfill these roles, social robots may offer similar benefits, particularly through processes of anthropomorphism. *Anthropomorphism/zoomorphism* describes the tendency to attribute human-like or animal-like characteristics to non-human entities (Epley et al., 2007). In this way, social robots transcend their mechanical nature; their gestures, speech, and responses function as symbolic acts that older adults interpret as signs of companionship and care (Gasteiger et al., 2021; Pu et al., 2019). These interactions foster emotional connections, allowing users to assign social meaning to the robot’s actions and perceive them as genuine sources of social support, which potentially reduces their feelings of loneliness (Bhandari & Baral Sapkota, 2023). Framing social robots in this way moves beyond a “technologically deterministic” perspective (Gallistl et al., 2024), emphasizing the importance of how older adults perceive and engage with technology. Despite this theoretical promise, the overall evidence remains inconclusive. A recent umbrella review found no significant pooled effects of social robots on depression, anxiety, or quality of life, although some individual studies reported positive outcomes (Nichol et al., 2024). A similar pattern may exist for loneliness, yet no meta-analysis has systematically quantified the effectiveness of social robots in reducing loneliness across diverse contexts (Balki et al., 2022); the only existing meta-analysis is limited to just two studies on robopets in care homes (Abbott et al., 2019). To address this gap, this study systematically evaluates the effectiveness of social robot interventions in reducing loneliness among older adults.

Hypothesis 1: Social robot interventions significantly reduce loneliness among older adults.

The effectiveness of social robot interventions is likely shaped by the social and cognitive contexts of the individuals who engage with them. Living arrangements in later-life could play a critical role in both the experience of loneliness and the reception of support. Evidence shows that older adults living alone or in institutional settings may benefit more from robot companionship, especially when human contact is limited or inconsistent (Lee et al., 2024). Cognitive status also could influence how older adults interpret and engage with robot interactions. Older adults with mild cognitive impairment or dementia often have distinct emotional and communicative needs, and may respond more positively to simplified, emotionally attuned, and predictable interactions—qualities frequently associated with social robots (Góngora Alonso et al., 2019; Van Assche et al., 2024). Cultural context may also influence intervention effectiveness. In collectivist societies, social robots may be seen as inadequate substitutes for human care, while in individualistic or technology-forward cultures, they are more readily accepted as supportive tools (Korn et al., 2021). Cross-national variation in digital literacy, care infrastructure, and policy further shapes how social robots are perceived and used (Bedaf et al., 2019). Finally, age may influence intervention outcomes, as younger-old adults often demonstrate greater technological adaptability and openness to engaging with social robots than their older counterparts (Yen et al., 2024).

Hypothesis 2: The effectiveness of social robots in reducing loneliness varies based on participant characteristics, including their living arrangement, cognitive status, country of residence, and age.

In addition to individual characteristics, features of the intervention itself may influence how effective social robots are in reducing loneliness. The type of social robot used plays a crucial role in shaping user experience and influencing effectiveness among older adults. Research suggests that pet-like robots tend to evoke emotional comfort and familiarity, making them particularly well-suited for soothing and companionship (Dosso et al., 2022). In contrast, humanoid robots and voice-based assistants often support more task-oriented or conversational interactions, which may appeal to users who prioritize responsiveness and verbal engagement (Zhang et al., 2024). The duration of social robot interventions may also affect outcomes, as longer-term engagements allow for the gradual development of emotional connection and trust between users and the robot (Whelan et al., 2018). Additionally, the year of publication may serve as a proxy for technological maturity and changing societal attitudes toward AI. More recent studies often report higher acceptance and greater efficacy, likely due to advances in robot capabilities, improved user interface design, and increased public exposure to AI technologies (Schroeder et al., 2023).

Hypothesis 3: The effectiveness of social robots in reducing loneliness varies based on intervention characteristics, including robot type, intervention duration, and year of publication.

## Method

### Data sources and search strategy

A comprehensive literature search was conducted from the inception of each database through October 1, 2024, using the following platforms: Scopus, EBSCO, and Web of Science, which contain multiple relevant datasets such as PsycINFO, PubMed, and CINAHL. We developed the retrieval strategy using Boolean logic, incorporating search terms related to social robots and loneliness among older adults, following the PICO’s principle (InJ et al., 2021), incorporating terms related to older adults, social robots, and loneliness. Full search terms for each database are available in the OSF repository: https://doi.org/10.17605/OSF.IO/W6T7X.

The study selection process was managed by Hubmeta (https://hubmeta.com/), a web-based platform designed to support systematic reviews through collaborative screening and data management. The authors independently screened and evaluated titles and abstracts for topic relevance and then reviewed full-text articles based on the inclusion and exclusion criteria to select the final articles. Full-text articles were then reviewed based on predefined inclusion and exclusion criteria. Inter-rater reliability was high (*κ* = 0.79, *p* < .001), and any disagreements were resolved through discussion and consensus. This systematic review has been registered with the PROSPERO International Prospective Register of Systematic Reviews under registration number: CRD42025639119. https://www.crd.york.ac.uk/PROSPERO/view/CRD42025639119

### Study selection

Eligibility criteria were pre-specified to ensure a systematic and objective selection process. Inclusion criteria included the following: (1) participants had to be older adults aged ≥50 years, and the total sample’s average age had to be 60 years or above; (2) older adults had to use robots designed to provide social or emotional interaction, excluding robot-assisted interventions such as surgical operations, gait training, exercise, or cognitive training; (3) the primary outcome had to be loneliness and quantitatively assessed.

Exclusion criteria included the following: (1) review articles, narrative studies, study protocols without results, and qualitative studies; (2) studies with a primary focus on behavioral effects related to mental health outcomes (e.g., technology acceptance, user behavior, or physical activity); (3) articles that did not contain sufficient statistical data for quantitative synthesis.

### Data extraction

Two independent reviewers conducted data extraction using predefined criteria to ensure consistency and rigor. A standardized database in Hubmeta was employed to systematically organize and document study information, reducing the risk of errors, omissions, and potential bias in the review process. Key variables collected included demographic information such as age range, minimum age, gender ratio, country, and living arrangements, as well as cognitive issues relevant to participant groups. Study-specific details such as the type of robot used, whether the robot was a pet robot, human/baby shape or personal voice assistant, duration of the intervention, and study design were also cataloged. Outcome measures were comprehensively recorded, including scales used to assess loneliness, sample sizes, and statistical data such as means and standard deviations for both pre-and post-intervention phases or between intervention and control groups. All data and analytic code used in this study are openly available in our OSF repository: https://doi.org/10.17605/OSF.IO/W6T7X.

### Quality assessment

The quality of the included studies was assessed using the Downs and Black Checklist (DBC) (Downs & Black, 1998). This checklist is a widely used tool for evaluating the methodological quality of quantitative research in both randomized and non-randomized healthcare intervention studies. The checklist assesses five key domains: study quality (reporting), external validity, internal validity (study bias), confounding and selection bias, and power of the study. The original DBC consists of 27 yes/no questions, providing both an overall quality score and individual section scores, with a maximum possible score of 28. However, specific items of the DBC are not applicable in the context of social robot interventions targeting loneliness, particularly due to the nature of psychosocial interventions and the ethical or practical constraints surrounding blinding procedures.

We excluded two items related to blinding (participants and outcome assessors) and one item on allocation concealment, as these were not applicable to psychosocial interventions involving social robots, where blinding is practically impossible, and allocation concealment is rarely used. This adjustment ensured that valid non-blinded or quasi-experimental designs were not unfairly penalized. As a result, the maximum possible score was adjusted to 24 instead of the original 28. This modified checklist allowed for a rigorous, context-appropriate evaluation of study quality, enhancing the credibility of the synthesized findings.

### Statistical analysis

The extracted data were imported into R software (version 4.3.2) for statistical analysis, which was conducted in four stages (R Foundation for Statistical Computing, Vienna, Austria). First, descriptive statistics were performed to summarize the characteristics of participants, intervention designs, and research methodologies. Second, small sample-corrected effect size estimates were calculated separately for studies with control groups and those without control groups, ensuring an accurate representation of intervention effects across study designs. Third, effect size estimates were synthesized across studies using robust variance estimation (RVE) for its ability to handle multiple, non-independent effect sizes within studies, a common issue in meta-analyses with nested data (Tipton, 2015), determining the overall effectiveness of social robots in reducing loneliness. Finally, moderator analyses were conducted to investigate participants’ characteristics and intervention features influencing the effectiveness of social robots on loneliness.

### Effect size synthesis

Effect sizes were calculated using Cohen’s *d* to quantify the impact of social robots on loneliness among older adults, utilizing the *metafor* package (version 4.6.0). For studies with control groups, effect sizes were computed as standardized mean differences with 95% confidence intervals (CIs), comparing loneliness outcomes between intervention and control groups (Lakens, 2013). For single-group studies employing pre- and post-intervention designs, standardized mean changes for repeated measures were used to assess within-group changes over time (Hopkins & Rowlands, 2024). Missing standard deviation values, which accounted for less than 24% of the dataset, were addressed using predictive mean matching (PMM) (Kleinke, 2017), implemented through the multivariate imputation by chained equations (*mice*) package in R (version 3.17.0). PMM generates realistic values without assuming a specific distribution, making it well-suited for continuous psychological outcomes such as loneliness. This method allowed the imputation of plausible values based on observed data patterns, maintaining the integrity of the synthesized estimates. Following the computation of individual effect sizes, these values were synthesized across studies to estimate the overall effectiveness of social robot interventions.

A meta-regression approach incorporating RVE, implemented using the *robumeta* package in R (version 2.1), was applied to synthesize the data while accounting for statistical dependence, which was introduced by studies reporting multiple effect sizes (Fisher & Tipton, 2015; Tanner‐Smith & Tipton, 2014). RVE has the advantage of requiring fewer assumptions about the sampling distribution of effect sizes and can estimate the covariance structure of dependent effect sizes without requiring additional statistical information (Pustejovsky & Tipton, 2022). Methodological recommendations suggest that RVE performs well even with smaller datasets, particularly when corrections for small sample sizes are applied, as was done in this review (Tipton, 2015). To further account for variance differences across studies and the non-independence of effect sizes within studies, hierarchical weights were applied in incorporating inverse-variance weighting while adjusting for clustering at the study level. The synthesized effect sizes provide a pooled estimate of the impact of social robots on loneliness, integrating findings from diverse study designs, intervention contexts, and participant characteristics. Given the diversity in study designs, we conducted a subgroup analysis separating studies with control groups from those without. This distinction is methodologically important, as controlled studies offer a more rigorous test of intervention effectiveness by accounting for potential confounding variables (Borenstein et al., 2021).

### Moderation analysis

Moderation analyses were conducted to examine sources of variability in intervention effectiveness, focusing on both participant characteristics and intervention design features. Participant characteristics included living arrangement, cognitive status, country of residence, and mean age of participants. The Living arrangement was treated as a categorical variable with four levels: their own home, nursing home, dementia care center, and hospital with living in one’s own home serving as the reference group (coded as 0). Cognitive status was coded dichotomously: individuals with no cognitive impairment (reference group, coded as 0) vs. those with some level of cognitive impairment or dementia. Another moderator was the country of study (e.g., United States, Japan, Turkey, China, Germany, South Korea, New Zealand, Taiwan, mixed). The United States was selected as the reference group, as it was the most frequently represented country in the dataset. Mean participant age was treated as a continuous variable to account for age-related differences in engagement with social robots.

Intervention characteristics included the type of social robot used, the duration of the intervention, and the year of study publication. (Tipton, 2015). Robot type (e.g., pet robot, humanoid, personal voice assistant) was included as a moderator, as different types of robots may elicit varying levels of engagement and emotional connection, potentially influencing their effectiveness in reducing loneliness. This variable was dummy-coded, with pet robots serving as the reference group (coded as 0). The duration of intervention was measured in weeks. The year of publication was treated as a continuous variable. Moderation analyses were conducted using RVE, which accommodates both continuous and categorical predictors while accounting for statistical dependence within studies (Tipton, 2015).

### Publication bias

Publication bias occurs when the published literature disproportionately reflects studies with significant results in a particular area. This often happens because studies yielding nonsignificant or negative results are less likely to be published, leading to an overestimation of effect sizes in the available literature (Dwan et al., 2013). Assessing publication bias is essential to determine whether the observed effect size may be inflated due to selective reporting. To assess potential publication bias in this review, a funnel plot was utilized to visually inspect the symmetry of effect size estimates plotted against their standard errors (Kepes et al., 2023). Subsequently, Egger’s test was conducted to statistically evaluate the presence of asymmetry, which may indicate publication bias (Egger et al., 1997). Consistent with the RVE framework employed in this study, Egger’s regression was implemented using the *robu()* function from the *robumeta* package (version 2.1) in R. In this approach, effect sizes were regressed on the inverse square root of their sampling variances to examine the association between effect size magnitude and precision. The *robu()* implementation also accounts for dependency among effect sizes by clustering on study-level units.

## Results

**Figure 1.**
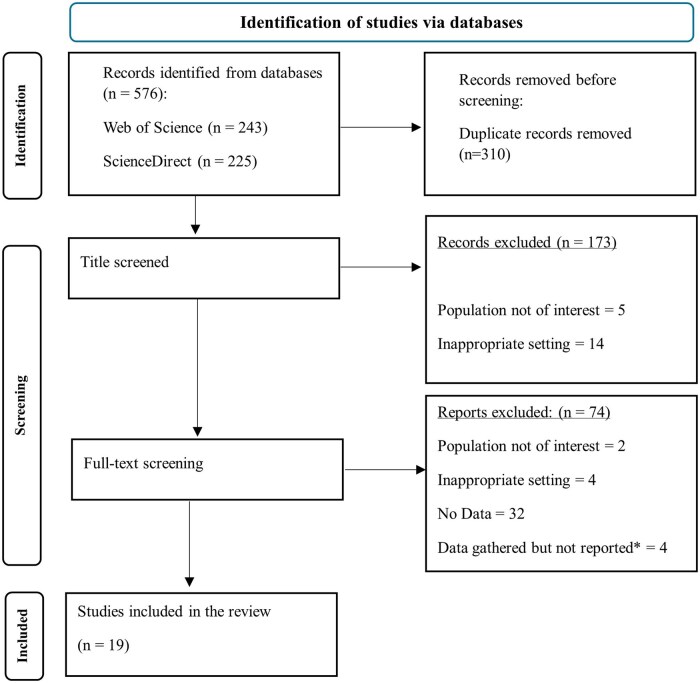
PRISMA diagram of search flow. ^*^Efforts were made to obtain missing statistical information by contacting study authors.

Figure 1 outlines the systematic process used for literature search and selection. Initially, 576 records were identified from database searches. After removing 310 duplicate entries, 266 unique records were screened based on their titles. Of these, 173 records were excluded, most commonly because the studies were qualitative, did not include older adult participants, or did not assess loneliness as an outcome, leaving 93 articles for full-text review. During the full-text review, of these 93, 74 records were excluded—primarily due to insufficient data for effect size calculation, lack of a social robot intervention, or failure to report pre- and post-intervention measures of loneliness. Attempts to retrieve missing data by contacting authors via email were unsuccessful.

Table 1 presents the characteristics of all studies included in this meta-analysis, encompassing a total sample of 1,083 participants. The studies varied in intervention type, participant demographics, and study settings. The included studies were published between 2003 and 2024. Detailed information is provided in Table 1.

**Table 1.** Study characteristics.

Study	**Intervention [control]** ^a^	Type of intervention	Sample size	Country	Mean age	Gender ratio (male %)	Living arrangement	Cognitive issues	Duration of intervention	Measurement scale
**Kanamori et al. (2003)**	AIBO	Pet robot	6	Japan	70.83	16.67	Nursing home	None	6 weeks	AOK
**Banks et al. (2008)**	AIBO [Control]	Pet robot	13 [13]	United States	-	-	Nursing home	None	8 weeks	UCLA
**Robinson et al. (2013)**	PARO [Control]	Pet robot	20 [20]	New Zealand	-	32.5	Nursing home	Some cognitive impairment	12 weeks	UCLA
Sidner et al. (2018)	Karen Virtual Agent	Personal voice assistant	18	United States	66	29.5	Their own home	None	4 weeks	UCLA
	**Reeti Robot**	Human-like robot	8							
**Chen et al. (2020)**	PARO	Pet robot	20	Taiwan	81.1	35	Nursing home	None	6 weeks	UCLA
**Fields et al. (2021)**	NAO	Human-like robot	15	United States	85.5	26.7	Nursing home	Some cognitive impairment	-	UCLA
Follmann et al. (2021)	TEMI-Euskirchen [Control]	Personal voice assistant	11 [11]	Germany	82.5	27.27	Nursing home	None	6 weeks	Hughes et al.
	**TEMI-Aachen [Control]**	Personal voice assistant	19 [9]	Germany	80.4	49.11	Nursing home	None	6 weeks	Hughes et al.
**Jones et al. (2021)**	Personal Voice Assistant (PVA)	Personal voice assistant	16	United States	85.2	31	Independent living facility	None	4 weeks	UCLA
Tkatch et al. (2021)	Animatronic Pet, Sample 1	Pet robot	168	United States	75.71	44.6	Their own home	None	-	UCLA
	**Animatronic Pet, Sample 2**	Pet robot	125	United States	75.68	43.2	Their own home	None	-	UCLA
**Chen et al. (2024a)**	PARO [Control]	Pet robot	26 [26]	China	79.8	57.7	Nursing home	Some cognitive impairment	8 weeks	UCLA
**Demirağ and Hintistan (2022)**	Robotic Cat [Control]	Pet robot	18 [18]	Turkey	67.22	50	Hospital	None	-	De Jong Gierveld
**Fogelson et al. (2022)**	Pet Robot	Pet robot	18	United States	89.6	11.11	Dementia Care Center	Dementia	3 weeks	UCLA
**Papadopoulos et al. (2022)**	CARESSES [Control]	Human-like robot	12 [11]	Mixed	79.5 [81.91]	41.66 [36.36]	Nursing home	None	2 weeks	UCLA
**Kim et al. (2023)**	Companion Robot	Personal voice assistant	186	South Korea	87.5	16.67	Their own home	None	12 weeks	UCLA
**Lim (2023)**	PIO [Control]	Pet robot	32 [33]	South Korea	75.39 [78.36]	6.5 [15.2]	Their own home	None	6 weeks	UCLA
**Chen et al. (2024b)**	PARO [Control]	Pet robot	58 [60]	Taiwan	81.78 [82.12]	39.7 [26.7]	Dementia Care Center	Dementia	6 weeks	UCLA
**Jones et al. (2024)**	Amazon Alexa	Personal voice assistant	34	United States	77	38	Independent living facility	None	12 weeks	UCLA
**Lei et al. (2024)**	Little Baby	Baby robot	44	China	77.18	22.22	Their own home	None	-	UCLA
**Yan et al. (2024)**	Amazon Alexa	Personal voice assistant	15	United States	85.2	26.66	Their own home	None	4 weeks	UCLA
			**1,083**							

*Note.* AIBO = Artificial Intelligence roBOt (Sony robot dog); PARO = personal robot modeled after a baby seal (used for therapeutic purposes); NAO = humanoid robot developed by SoftBank robotics; TEMI = personal assistant robot with mobility and voice interaction; CARESSES = culture-aware robots and environmental sensor systems for elderly support (EU–Japan project); PIO = Pepper-Inspired Older adult robot (NAO variant); UCLA = University of California, Los Angeles (used in the naming of the UCLA Loneliness Scale).

a Values in brackets represent data for the control group in studies that included a control condition.

### Publication bias and quality assessment

**Figure 2.**
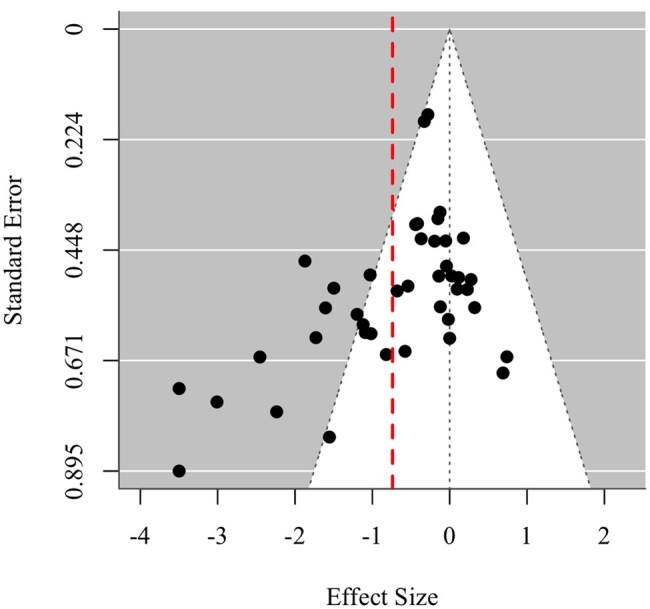
Funnel plot for assessing publication bias. The dashed vertical line shows the pooled effect size. The triangular region represents the expected distribution of studies in the absence of bias, with points outside this region potentially indicating asymmetry or bias.

Publication bias was assessed using a funnel plot (Figure 2), which plotted the observed effect size estimates against their standard errors. In an unbiased sample, studies would be symmetrically distributed around the pooled effect size. However, the plot showed a clustering of positive effects, suggesting asymmetry. The red dashed line represents the pooled effect size. The distribution of the effect size estimates revealed moderate asymmetry, suggesting potential publication bias. To statistically evaluate this asymmetry, Egger’s regression test was conducted using a RVE model, incorporating precision [1/sqrt (Variance)] as a moderator. The results revealed a significant intercept (*β* = −0.825, *SE* = 0.284, *p* = <.01), indicating potential bias in the distribution of effect sizes. This suggests that smaller studies with non-significant or negative results may be underrepresented.

However, it is important to interpret the observed asymmetry in light of the intervention context. Social robots are designed to reduce loneliness, and it is unlikely that their presence would dramatically increase loneliness. While small increases in loneliness might occur in certain circumstances—perhaps due to unmet expectations or limited interaction quality—extreme increases in loneliness are unlikely, given the nature and intent of the intervention. The clustering of studies with negative effect sizes (reduced loneliness) to the left of the center line likely reflects the intended effect of the intervention, while the lack of extreme positive effect sizes (increased loneliness) supports the argument that such outcomes are unlikely in real-world settings. Therefore, while Egger’s test indicates asymmetry, caution is needed in interpreting this as definitive evidence of bias, as it may not fully account for the contextual constraints on the intervention’s effects (Sterne et al., 2011).

The methodological quality of the included studies, assessed using a structured checklist, varied across studies. As shown in Table 2, quality scores ranged from 14 to 21 out of a maximum of 24. Fifteen of the 19 studies scored above 16, representing at least 70% of the total possible points, indicating moderate to high quality. Given the limited number of available studies, separate meta-analyses based on study quality were not conducted. Instead, study quality was taken into account during interpretation to provide a balanced understanding of the findings.

**Table 2.** Quality assessment of studies by subscale: Score (% of the maximum achievable score).

Study	Reporting (10)	External validity (3)	Bias (5)	Confounding (5)	Power (1)	Total (24)
**Kanamori et al. (2003)**	7 (70)	1 (33)	4 (80)	2 (40)	1 (33)	15 (62.5)
**Banks et al. (2008)**	5 (50)	1 (33)	5 (100)	3 (60)	0 (0)	14 (58.3)
**Robinson et al. (2013)**	8 (80)	1 (33)	5 (100)	3 (60)	1 (33)	18 (75)
**Sidner et al. (2018)**	7 (70)	1 (33)	4 (80)	4 (80)	1 (33)	17 (70.8)
**Chen et al. (2020)**	9 (90)	1 (33)	4 (80)	2 (40)	1 (33)	17 (70.8)
**Fields et al. (2021)**	9 (90)	1 (33)	4 (80)	3 (60)	1 (33)	17 (70.8)
**Follmann et al. (2021)**	8 (80)	1 (33)	5 (100)	2 (40)	1 (33)	17 (70.8)
**Jones et al. (2021)**	7 (70)	1 (33)	4 (80)	2 (40)	1 (33)	15 (62.5)
**Tkatch et al. (2021)**	7 (70)	1 (33)	4 (80)	3 (60)	1 (33)	16 (66.6)
**Demirağ and Hintistan (2022)**	7 (70)	0 (0)	5 (100)	4 (80)	0 (0)	16 (66.6)
**Fogelson et al. (2022)**	10 (100)	1 (33)	4 (80)	2 (40)	1 (33)	18 (75)
**Papadopoulos et al. (2022)**	9 (90)	1 (33)	5 (100)	4 (80)	1 (33)	20 (83.3)
**Lim (2023)**	9 (90)	1 (33)	5 (100)	3 (60)	1 (33)	19 (79.16)
**Kim et al. (2023)**	10 (100)	1 (33)	4 (80)	3 (60)	1 (33)	19 (79.16)
**Chen et al. (2024b)**	10 (100)	1 (33)	5 (100)	4 (80)	1 (33)	21 (87.5)
**Chen et al. (2024a)**	9 (90)	1 (33)	4 (80)	4 (80)	1 (33)	19 (79.16)
**Jones et al. (2024)**	7 (70)	1 (33)	4 (80)	2 (40)	1 (33)	15 (62.5)
**Lei et al. (2024)**	8 (80)	1 (33)	4 (80)	3 (60)	1 (33)	17 (70.8)
**Yan et al. (2024)**	8 (80)	1 (33)	4 (80)	3 (60)	1 (33)	17 (70.8)

### Meta-analytic results of the effectiveness of social robots

**Figure 3.**
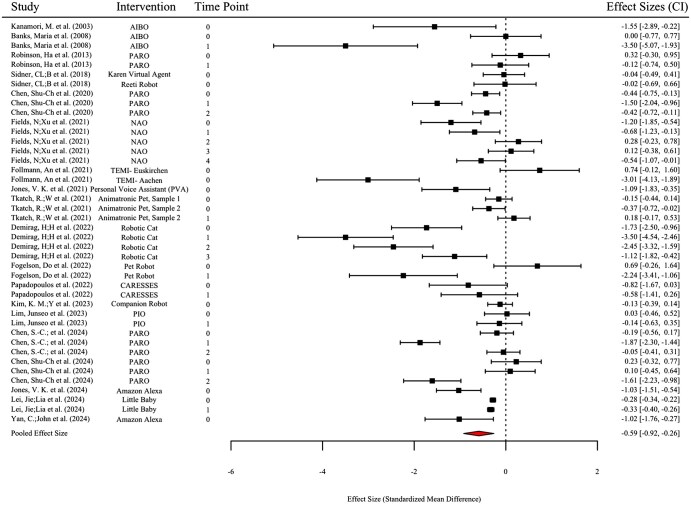
Forest plot. Studies are listed by year of publication and time point. Horizontal lines represent 95% CI. Squares indicate the effect sizes (Cohen’s *d*) for individual studies, with the size proportional to the study’s relative weight. The diamond represents the pooled effect size from the random-effects meta-analysis. Columns display the study name, intervention name, time point (0 = pre-intervention, 1+ = post-intervention/s), and effect sizes with CI.

The meta-analysis evaluated the effectiveness of social robots in reducing loneliness among older adults, based on 42 effect sizes drawn from 19 studies. Figure 3 presents the forest plot, which visually depicts the effect size estimates for individual studies and their CI. Moreover, Table 3 presents a statistical summary of these findings, including pooled effect sizes, standard errors, CI, and heterogeneity estimates. The overall pooled effect size was −0.590 (*p* < .01), indicating a statistically significant and large reduction in loneliness, based on Brydges’ (2019) criteria for interpreting effect sizes in aging research.

**Table 3.** Meta-analysis results: effectiveness of social robot interventions on loneliness.

	*N/K*	*d*	*SE*	*p*-Value	95% CI	*τ*²
**Total**	19/42	−0.590	0.153	.002^**^	[−0.919, −0.261]	0.236
**With control group**	8/20	−0.878	0.349	.042^*^	[−1.71, 0.042]	1.008
**Without control group**	11/22	−0.403	0.096	.005^**^	[−0.639, −0.167]	0.042

*Note. N=* number of studies; *K=* number of effect sizes; *d*= the pooled effect size, expressed as Cohen’s *d* (negative values indicate a reduction in loneliness); *SE*= standard error of the effect size; 95% CI *= *95% confidence interval; *= p<.05; **= p<.01..

Between-study variability was moderate, indicating that the impact of social robots on loneliness is not uniform but depends on how the intervention is implemented. Subgroup analysis revealed that study design played a significant role in shaping the outcome. Specifically, studies employing control groups (8 studies, 20 effect sizes) showed a larger, stronger, and significant reduction in loneliness, suggesting that rigorously tested interventions may yield stronger effects. In contrast, studies without a control group (11 studies, 22 effect sizes) demonstrated a smaller, though still significant, reduction in loneliness.

### Moderation analysis

Table 4 summarizes the results of the moderation analyses, examining whether participant and intervention characteristics influenced the effectiveness of social robots. With respect to participant characteristics, the living arrangement significantly moderated intervention outcomes. Participants residing in institutional settings—including nursing homes or independent living facilities, dementia care centers, and hospitals—experienced significantly greater reductions in loneliness compared to those living in their own homes. The country in which the study was conducted emerged as a significant moderator. Studies conducted in Japan and Turkey reported larger reductions in loneliness compared to studies from the United States, while studies from New Zealand demonstrated smaller reductions. No significant differences were found for studies conducted in China, Germany, South Korea, Taiwan, or in mixed-country samples. Moreover, Cognitive status did not significantly moderate intervention outcomes; participants with dementia or cognitive impairments experienced effects similar to those without impairments. Mean participant age was not a significant moderator.

**Table 4.** Moderator meta-regression analysis for participant and intervention characteristics.

Moderator	*N/K*	*β*	*df*	95% CI
**Living arrangement (*ref:* their own home)**				
**Nursing home or independent facility**	10/22	−0.393**	8.54	[−0.760, −0.025]
**Dementia Care Center**	2/5	−0.468**	1.82	[−0.889, −0.047]
**Hospital**	1/4	−1.887***	4.55	[−2.241, −1.534]
**Cognitive issues (*ref:* None)**				
**Dementia or some cognitive impairment**	5/15	0.297	7.26	[−0.317, 0.910]
**Country (*ref:* United States)**				
**China**	2/5	0.115	1.77	[−0.606, 0.835]
**Germany**	1/2	−0.438	1.18	[−17.144, 16.269]
**Japan**	1/1	−1.083***	4.38	[−1.456, −0.710]
**South Korea**	2/3	0.390	1.45	[−0.513, 1.293]
**New Zealand**	1/2	0.572**	4.38	[0.199, 0.945]
**Taiwan**	2/6	−0.247	1.96	[−0.873, 0.378]
**Turkey**	1/4	−1.627***	4.85	[−2.062, −1.192]
**Mixed**	1/2	−0.226	4.39	[−0.599, 0.147]
**Robot type (*ref:* pet robot)**				
**Humanoid (human/baby shape)**	4/10	0.327	4.10	[−0.408, 1.062]
**Personal voice assistant**	6/7	0.013	8.97	[−1.003, 1.030]

*Note. N* = number of studies; *K* = number of effect sizes; *β* = estimate represents the change in 𝑑 associated with each predictor relative to the reference group; *df* = degrees of freedom; 95% CI = 95% confidence interval.

** *p* < .01,

*** *p* < .001.

Regarding intervention characteristics, the type of social robot used did not significantly influence intervention effectiveness. Specifically, neither humanoid robots nor voice assistants produced significantly different effects compared to pet robots. Lastly, neither intervention duration nor publication year significantly moderated intervention outcomes.

## Discussion

The findings of this meta-analysis provide robust evidence that social robots are effective tools for reducing loneliness among older adults, demonstrating a large overall effect size. This finding reinforces the growing recognition of social robots not merely as assistive devices but as potential contributors to psychosocial well-being in aging populations.

The impact of study design on intervention effectiveness is particularly noteworthy. Studies employing control groups demonstrated stronger reductions in loneliness than pre-post designs, suggesting that more robust evaluation methods are better equipped to capture the true effects of social robot interventions. This distinction is especially important in the context of loneliness, where outcomes may be influenced by participant expectations, the novelty of interacting with a robot, or natural changes over time (Freedland et al., 2011). This finding reinforces the importance of methodological rigor in social robot research and aligns with broader intervention science standards, including Cochrane guidelines, which emphasize randomized controlled trials as the gold standard for establishing causal effects (Johnson et al., 2015).

The role of participant characteristics further illustrates the contextual factors that shape the effectiveness of social robot interventions. Living arrangement significantly moderated outcomes, with older adults residing in institutional settings, such as nursing homes, independent living facilities, dementia care centers, and hospitals, experiencing larger reductions in loneliness compared to those living independently. As Goffman (1961) described, institutional settings function as *total institutions*, structured environments where individuals are cut off from broader society and lead enclosed, formally administered lives. Within such contexts, opportunities for social interaction are limited (Neves et al., 2019), and social robots could be particularly well-positioned to meet unmet relational needs. Country of residence also significantly moderated intervention effects, with studies conducted in Japan and Turkey reporting stronger reductions in loneliness compared to the United States, while studies from New Zealand showed smaller effects. These variations likely reflect broader cultural attitudes toward aging, caregiving, and technology adoption. For instance, Japan’s cultural normalization of robotic technologies (Šabanović, 2014) may foster greater acceptance, while Turkey’s communal caregiving norms could shape positive perceptions of supplemental companionship (Çonka et al., 2025).

In contrast, cognitive status and age did not significantly influence intervention outcomes. The non-significance of cognitive impairment challenges assumptions that social robots are less effective among individuals with cognitive decline, suggesting instead that consistent, engaging interactions may benefit older adults across cognitive profiles. Similarly, the absence of a significant moderating effect for mean age suggests that the benefits of social robots are broadly applicable across the older adult age spectrum. This finding challenges the notion that only younger individuals or those more technologically savvy derive benefits from these interventions.

Regarding intervention characteristics, the type of social robot, duration of intervention, and year of publication did not significantly influence effectiveness of social robots. The lack of moderation by robot type is particularly striking, as it counters much of the design-focused discourse emphasizing the necessity of complex features (Broadbent, 2017). Our findings suggest that even brief, simple interventions may produce meaningful benefits and that the effectiveness of social robots has remained stable despite technological advancements over time. Taken together, these results shift attention away from robot sophistication toward the relational dynamics between older adults and robots, emphasizing that the social meaning users assign to the interaction and trust, not the robot’s technical capabilities, are critical factors underlying intervention success (Ghafurian et al., 2022).

### Ethical and policy implications

While this meta-analysis affirms the promise of social robots in reducing loneliness, especially in institutional contexts, their implementation must be approached with attention to structural inequality and ethical care. Without deliberate equity-oriented strategies, such technologies may disproportionately benefit those with higher socioeconomic status or digital literacy and reinforce social disparities among older adults (Rubeis et al., 2022). Concerns surrounding privacy, informed consent, and algorithmic fairness require thoughtful and inclusive design, particularly for older adults with cognitive impairments or limited digital literacy, who are more vulnerable to misunderstanding, exclusion, or exploitation in human-robot interactions (Ho, 2023). Finally, it is essential to ensure that social robots complement rather than replace human relationships, safeguarding against the risk of an “alone together” scenario, described by Wood (2012), in which individuals are physically connected to technology but emotionally disconnected from others. Future policies should prioritize culturally responsive, affordable, and user-centered robotic tools and support participatory design processes that include older adults in shaping the technologies intended for them.

### Limitations

Despite providing valuable insights into the effectiveness of social robots in reducing loneliness among older adults, this meta-analysis has limitations that should be acknowledged. First, publication bias was detected through Egger’s test and funnel plot analysis, indicating a potential overestimate the true impact of social robots on loneliness. While efforts were made to address this issue by using RVE, the asymmetry observed suggests caution in generalizing the results. Additionally, most studies were conducted in high-income countries, limiting the applicability of findings to low- and middle-income settings where access to social robots may differ significantly.

### Future research directions

Several important avenues for future research emerge from this meta-analysis. First, there is a pressing need for longitudinal studies to assess the sustainability of social robot interventions over time. While short-term reductions in loneliness are evident, it remains unclear whether these effects persist in the long term or diminish as novelty wears off. Future research should also prioritize the inclusion of underrepresented populations, particularly individuals from diverse socioeconomic, cultural, and ethnic backgrounds. Much of the existing research disproportionately represents homogeneous, advantaged groups, limiting the generalizability of findings. Understanding how social robots function across varying social, cultural, and structural contexts will be essential to ensuring these interventions are effective and equitable. In addition, we encourage more interdisciplinary approaches that bridge sociology, psychology, gerontology, and engineering to develop socially intelligent robots capable of addressing complex psychological needs. Mixed-methods research will also be invaluable in capturing subjective experiences with social robots, particularly how users interpret, adapt to, and derive meaning from these interactions. Furthermore, future studies should move beyond focusing solely on robot appearance or form and instead explore how interaction strategies, such as conversation quality, responsiveness, and adaptability, can be optimized to maximize psychosocial benefits.

To facilitate higher methodological quality and transparency in future studies, we propose a Checklist for Assessing Robot Effectiveness (CARE) for mental health outcomes. Developed to address key reporting gaps identified in this review, CARE is applicable across age groups and a range of psychological outcomes. It covers essential elements such as participant demographics (e.g., age, gender, socioeconomic background), robot type and features, detailed interaction protocols, measurement tools, control conditions (if applicable), and cultural or accessibility considerations. Specific items tailored to older adult populations, such as cognitive status and living arrangements, are also included. Additionally, CARE highlights the importance of reporting adherence rates, participant feedback, and complete statistical data, including pre- and post-intervention results and attrition rates. The full checklist is available in the Supplementary Material (see online supplementary material). We strongly encourage future researchers to adopt CARE to improve reporting consistency, facilitate cross-study comparisons, and strengthen the overall quality of evidence in this growing field.

## Supplementary Material

gnaf219_Supplementary_Data

## Data Availability

This study was preregistered on PROSPERO (CRD42025639119). All data, coding materials, and analytic scripts used in this meta-analysis are available from the authors upon request. We adhered to PRISMA guidelines and have included the PRISMA checklist and flow diagram in the Supplementary Material (see online supplementary material).
