# Supplementary material for: Wired for companionship: a meta-analysis on social robots filling the void of loneliness in later life
Source: Gerontologist. 2025 Sep 30;65(12):gnaf219. doi: 10.1093/geront/gnaf219 (PMC12598933; doi:10.1093/geront/gnaf219)
Supplement: gnaf219_Supplementary_Data [file gnaf219_supplementary_data.zip › Mehrabi_Ghezelbash_Suppl.docx]

Supplementary Material

| **Section and Topic** | **Item #** | **Checklist item** | **Location where item is reported** |
| --- | --- | --- | --- |
| **TITLE** | | |  |
| Title | 1 | Identify the report as a systematic review. | 1 |
| **ABSTRACT** | | |  |
| Abstract | 2 | See the PRISMA 2020 for Abstracts checklist. | 2 |
| **INTRODUCTION** | | |  |
| Rationale | 3 | Describe the rationale for the review in the context of existing knowledge. | 3-7 |
| Objectives | 4 | Provide an explicit statement of the objective(s) or question(s) the review addresses. | 5-7 |
| **METHODS** | | |  |
| Eligibility criteria | 5 | Specify the inclusion and exclusion criteria for the review and how studies were grouped for the syntheses. | 9 |
| Information sources | 6 | Specify all databases, registers, websites, organisations, reference lists and other sources searched or consulted to identify studies. Specify the date when each source was last searched or consulted. | 7&8 |
| Search strategy | 7 | Present the full search strategies for all databases, registers and websites, including any filters and limits used. | 8 |
| Selection process | 8 | Specify the methods used to decide whether a study met the inclusion criteria of the review, including how many reviewers screened each record and each report retrieved, whether they worked independently, and if applicable, details of automation tools used in the process. | 7&8 |
| Data collection process | 9 | Specify the methods used to collect data from reports, including how many reviewers collected data from each report, whether they worked independently, any processes for obtaining or confirming data from study investigators, and if applicable, details of automation tools used in the process. | 9&10 |
| Data items | 10a | List and define all outcomes for which data were sought. Specify whether all results that were compatible with each outcome domain in each study were sought (e.g. for all measures, time points, analyses), and if not, the methods used to decide which results to collect. | 9 |
|  | 10b | List and define all other variables for which data were sought (e.g. participant and intervention characteristics, funding sources). Describe any assumptions made about any missing or unclear information. | 9&10 |
| Study risk of bias assessment | 11 | Specify the methods used to assess risk of bias in the included studies, including details of the tool(s) used, how many reviewers assessed each study and whether they worked independently, and if applicable, details of automation tools used in the process. | 10 |
| Effect measures | 12 | Specify for each outcome the effect measure(s) (e.g. risk ratio, mean difference) used in the synthesis or presentation of results. | 11 |
| Synthesis methods | 13a | Describe the processes used to decide which studies were eligible for each synthesis (e.g. tabulating the study intervention characteristics and comparing against the planned groups for each synthesis (item #5)). | 11&12 |
|  | 13b | Describe any methods required to prepare the data for presentation or synthesis, such as handling of missing summary statistics, or data conversions. | 11&12 |
|  | 13c | Describe any methods used to tabulate or visually display results of individual studies and syntheses. | 11&12 |
|  | 13d | Describe any methods used to synthesize results and provide a rationale for the choice(s). If meta-analysis was performed, describe the model(s), method(s) to identify the presence and extent of statistical heterogeneity, and software package(s) used. | 11&12 |
|  | 13e | Describe any methods used to explore possible causes of heterogeneity among study results (e.g. subgroup analysis, meta-regression). | 11-13 |
|  | 13f | Describe any sensitivity analyses conducted to assess robustness of the synthesized results. | 11&12 |
| Reporting bias assessment | 14 | Describe any methods used to assess risk of bias due to missing results in a synthesis (arising from reporting biases). | 13&14 |
| Certainty assessment | 15 | Describe any methods used to assess certainty (or confidence) in the body of evidence for an outcome. | NA |
| **RESULTS** | | |  |
| Study selection | 16a | Describe the results of the search and selection process, from the number of records identified in the search to the number of studies included in the review, ideally using a flow diagram. | 14 |
|  | 16b | Cite studies that might appear to meet the inclusion criteria, but which were excluded, and explain why they were excluded. | NA |
| Study characteristics | 17 | Cite each included study and present its characteristics. | 16&17 |
| Risk of bias in studies | 18 | Present assessments of risk of bias for each included study. | 19 |
| Results of individual studies | 19 | For all outcomes, present, for each study: (a) summary statistics for each group (where appropriate) and (b) an effect estimate and its precision (e.g. confidence/credible interval), ideally using structured tables or plots. | 20 |
| Results of syntheses | 20a | For each synthesis, briefly summarise the characteristics and risk of bias among contributing studies. |  |
|  | 20b | Present results of all statistical syntheses conducted. If meta-analysis was done, present for each the summary estimate and its precision (e.g. confidence/credible interval) and measures of statistical heterogeneity. If comparing groups, describe the direction of the effect. | 20 |
|  | 20c | Present results of all investigations of possible causes of heterogeneity among study results. | 20-22 |
|  | 20d | Present results of all sensitivity analyses conducted to assess the robustness of the synthesized results. | 21 |
| Reporting biases | 21 | Present assessments of risk of bias due to missing results (arising from reporting biases) for each synthesis assessed. | 14&15 |
| Certainty of evidence | 22 | Present assessments of certainty (or confidence) in the body of evidence for each outcome assessed. | NA |
| **DISCUSSION** | | |  |
| Discussion | 23a | Provide a general interpretation of the results in the context of other evidence. | 23-25 |
|  | 23b | Discuss any limitations of the evidence included in the review. | 26 |
|  | 23c | Discuss any limitations of the review processes used. | 26 |
|  | 23d | Discuss implications of the results for practice, policy, and future research. | 26&27 |
| **OTHER INFORMATION** | | |  |
| Registration and protocol | 24a | Provide registration information for the review, including register name and registration number, or state that the review was not registered. | 8 |
|  | 24b | Indicate where the review protocol can be accessed, or state that a protocol was not prepared. | 8 |
|  | 24c | Describe and explain any amendments to information provided at registration or in the protocol. | NA |
| Support | 25 | Describe sources of financial or non-financial support for the review, and the role of the funders or sponsors in the review. | NA |
| Competing interests | 26 | Declare any competing interests of review authors. | NA |
| Availability of data, code and other materials | 27 | Report which of the following are publicly available and where they can be found: template data collection forms; data extracted from included studies; data used for all analyses; analytic code; any other materials used in the review. | NA |

*From:*  Page MJ, McKenzie JE, Bossuyt PM, Boutron I, Hoffmann TC, Mulrow CD, et al. The PRISMA 2020 statement: an updated guideline for reporting systematic reviews. BMJ 2021;372:n71. doi: 10.1136/bmj.n71. This work is licensed under CC BY 4.0. To view a copy of this license, visit <https://creativecommons.org/licenses/by/4.0/>

**Checklist for Assessing Robot Effectiveness (CARE) for Mental Health Outcomes**

| Item | | Description | Reported? |
| --- | --- | --- | --- |
| 1 | Participant Demographics | Provide age range, mean age, gender distribution, and socioeconomic background (including education level, marital status, income if available). | ☐ Yes ☐ No |
| 2 | Cognitive Status (*just for older adults*) | Clearly report participants’ cognitive health status (no impairment, mild cognitive impairment, dementia). | ☐ Yes ☐ No |
| 3 | Living Arrangement (*just for older adults*) | Specify whether participants live at home, in nursing homes, assisted living facilities, dementia care centers, or hospitals. | ☐ Yes ☐ No |
| 4 | Intervention Setting | Describe the physical and social context (e.g., individual sessions, group activities, institutional setting, community-based setting). | ☐ Yes ☐ No |
| 5 | Robot Type & Features | Fully describe the robot type (e.g., pet robot, humanoid, personal voice assistant), along with key features such as mobility, speech capabilities, tactile or sensory abilities, and interactivity. | ☐ Yes ☐ No |
| 6 | AI Advancement Level | Clearly specify the robot's level of AI sophistication (e.g., non-AI/static, rule-based interaction, adaptive learning, autonomous decision-making). | ☐ Yes ☐ No |
| 7 | Robot Interaction Protocol | Provide a detailed description of interaction protocols, including session frequency, session duration, type of interaction (e.g., conversation, physical touch, games), and any personalization elements. | ☐ Yes ☐ No |
| 8 | Duration of Intervention | Report the total length of the intervention (in weeks) and session frequency (e.g., weekly, daily). | ☐ Yes ☐ No |
| 9 | Measurement Tools for Mental Health Outcomes | Specify and justify the measurement tools used to assess mental health outcomes (e.g., loneliness, depression, anxiety), including information on scoring methods and psychometric properties. | ☐ Yes ☐ No |
| 10 | Secondary Outcome Measures | Report any additional psychosocial or cognitive outcomes assessed (e.g., well-being, stress, social support). | ☐ Yes ☐ No |
| 11 | Control Group Details (if applicable) | Clearly describe control group conditions (e.g., usual care, alternative intervention, waitlist). | ☐ Yes ☐ No |
| 12 | Adherence & Engagement Reporting | Provide detailed information on participant engagement, including attendance rates, interaction duration, dropout rates, and reasons for attrition. | ☐ Yes ☐ No |
| 13 | Participant Feedback/User Experience | Include qualitative and/or quantitative feedback from participants regarding their experience with the robot and overall satisfaction. | ☐ Yes ☐ No |
| 14 | Cultural Adaptation | Report any adaptations made to the intervention or robot features to fit the cultural, linguistic, or social context of the participant population. | ☐ Yes ☐ No |
| 15 | Accessibility Considerations | Address accessibility aspects such as cost, ease of use, digital literacy requirements, and whether the intervention is feasible for participants to access or purchase independently. | ☐ Yes ☐ No |
| 16 | Ethical Approval & Consent | Clearly state ethical approval details and describe the informed consent process. | ☐ Yes ☐ No |
| 17 | Pre-Intervention Statistics | Report means, standard deviations (SD), and sample sizes (n) for all outcome measures before the intervention, separately for each group. | ☐ Yes ☐ No |
| 18 | Post-Intervention Statistics | Report means, SDs, and sample sizes (n) for all outcome measures after the intervention, separately for each group. | ☐ Yes ☐ No |
| 19 | Between-Group Comparison Data (if applicable) | For studies with control groups, report statistical comparisons between groups (mean differences, t-values, p-values, confidence intervals, effect sizes). | ☐ Yes ☐ No |
| 20 | Within-Group Comparison Data | For pre-post designs, report paired statistical analyses (e.g., t-tests), mean differences, SDs, confidence intervals, and effect sizes. | ☐ Yes ☐ No |
| 21 | Missing Data & Attrition Reporting | Provide complete information on missing data, dropout rates, reasons for attrition, and how missing data were handled in the analysis. | ☐ Yes ☐ No |
